# Perceived social support attenuates increased hostile reactions to traumatic threat

**DOI:** 10.1002/jclp.23567

**Published:** 2023-07-12

**Authors:** S. C. Napolitano, C. E. Balling, I. Peckinpaugh, D. B. Samuel, S. P. Lane

**Affiliations:** 1 Department of Psychological Sciences, Purdue University, West Lafayette, Indiana, USA; 2 Department of Psychological Sciences, University of Missouri, Columbia, Missouri, USA

**Keywords:** distress, hostility, perceived social support, social isolation, trauma

## Abstract

**Objective:**

Extant literature has seldom examined the naturalistic role of reaction to threat on downstream emotional distress while also considering buffers, such as perceived social support, to acute negative mental health outcomes. The present study examined how trauma symptoms, in reaction to a global stressor, predicted increased psychological distress via elevated emotional hostility and whether perceived social support modified such effects. We predicted *a priori* that increased exposure to trauma would be associated with increased hostility and global psychological distress, but that this path would be attenuated by greater levels of perceived social support, as individuals who report greater support exhibit greater emotional coping.

**Methods:**

We recruited 408 adults from a large university in the Midwestern United States to participate in a survey assessing past-week trauma, hostility, distress, and perceived social support following the initial COVID-19 lockdown. The survey was conducted in March 2020, directly after strict shelter-in-place orders were locally mandated. To test our hypotheses, we employed a moderated mediation analysis approach.

**Results:**

Results demonstrate that higher trauma predicted increased hostility, which in turn predicted increased distress, and trauma predicted distress via hostility (an indirect effect). As hypothesized, higher perceived social support attenuated the association between trauma and hostility.

**Conclusion:**

Results support a hostile emotional pathway that may increase distress in the context of increased traumatic impact; however, social support likely buffers these effects, particularly in the face of new or novel threats and stressors. Findings suggest broad application for understanding the relation between the introduction of stressors, psychological distress, and social support.

## 1 | INTRODUCTION

Research suggests a well-established causal path between the experience of traumatic impact from an aversive event and increased psychological distress. Specifically, increased hyperarousal, intrusive thoughts, and avoidance related to threat contribute downstream to amplified anxiety, depression, and stress symptoms (Morrill et al., 2008). Fortunately, perceived social support is a robust mitigator of the deleterious effects of increased stress, especially for acute, immediate stressors (Arnberg et al., 2012; Clark, 2003; Cohen et al., 1986). Social support has also been found to alleviate immediate physical stressors such as pain (Brown et al., 2003). An abundant literature provides evidence for social support’s role in buffering the effects of stress (Catabay et al., 2019; Hughes et al., 2020; Olstad et al., 2001). According to social support theory, higher perceived levels of social support should directly attenuate immediate stress responses (Clark, 2003; Schwerdtfeger & Schlagert, 2011) and modify the relationship between reactions to threat and consequential psychological distress (Arnberg et al., 2012; Cohen et al., 1986). Research indicates that increased perceived social support uniquely buffers against the pernicious effects of stress, such that the more perceived social support an individual reports, the more that support buffers against significant psychological distress (Olstad et al., 2001; Wang et al., 2014). Social support has even been found to reduce somatic responses through mediating the relationship between trauma exposure and posttraumatic stress disorder (PTSD) symptoms (Catabay et al., 2019).

One idea of why this buffering effect may occur is that perceived social support bolsters one’s sense of self-efficacy and imparts a sense that others close to the individual are available to “share the burden” (Norris & Kaniasty, 1996). In turn, this shield helps curb downstream effects of increased hostility that otherwise appear to extrapolate, including increased daily stress and symptoms of anxiety and depression. This is relevant, as these symptoms, when not addressed, can contribute to long-term patterns and potentially clinical levels of psychopathology (Catabay et al., 2019; Heponiemi et al., 2006).

Aside from the link between traumatic impact and increased distress (Lahav, 2020), one particularly salient difference that has yet to be addressed in the literature is the difference in reactions to threat and subsequent psychological distress. In the face of an unpredictable, imminent threat, individuals are likely to exhibit a multitude of varied responses, such that general fear-driven appraisals yield differential, specific emotional reactions to threat. One such reaction is increased hostility and anger directed at the threat, which then directly informs the likelihood of behavioral aggression (MacLaren et al., 2010).

## 2 | THREAT AND HOSTILITY

Although reaction to threat (i.e., heightened arousal) may call upon both approach and withdraw motivations on some level, tension between approach and avoidance orientations is typically viewed categorically. Whereas individuals may feel ambivalence toward both approach and avoidance stimuli, one orientation “wins” over the other to direct attentional resources and consequential behavior (Appraisal-Tendency Theory; Lerner & Keltner, 2001). Typically, a greater danger appraisal of the threat leads to a more pessimistic prediction of outcome, and consequently, greater precautions taken against the threat. Conversely, a less dangerous appraisal of the threat may allow individuals to allocate attentional resources to themselves, empowering them to take potentially fewer precautions against the threat. Put simply, an individual’s opinion regarding the danger of the threat may impact an outcome, such as the spread of a disease given the behavioral choices made from the appraisal (Tyson et al., 2020) and awareness (Li et al., 2020) of the threat. In the case of an overwhelming threat that leads to trauma, individuals are likely to experience subsequent increased psychological distress in response to the weight of the threat (i.e., a traumatic reaction). However, those who are more aggressive or view themselves as more capable of taking on the threat may report an increase in feelings of hostility as attentional resources are gathered to confront the threat (e.g., MacLaren et al., 2010; Twenge et al., 2001). Here, hostility reflects a negative attitude toward an outside stimulus, such as another person, that reflects an unfavorable appraisal of that stimulus (Berkowitz, 1993). Importantly, hostility itself is delineated from anger in its externalization. Specifically, Speilberger (1988) states that hostility reflects a complex, dynamic emotion that motivates aggressive and vindictive behavior. Indeed, researchers have linked heightened levels of hostility with approach orientation in the presence of threat (Lerner & keltner, 2001; MacLaren et al., 2010) and other evidence links social threat with increased hostility (Twenge et al., 2001). Although indirect, this literature on responses to both manageable and overwhelming threats serves as a parallel to inform likely consequences to traumatic reactions, given that trauma itself is a sustained, high-arousal state of feeling threatened.

## 3 | HOSTILITY, DISTRESS, AND PERCEIVED SOCIAL SUPPORT

A relatively small body of research constitutes what we know about increased hostility and downstream emotional distress, but the preponderance of evidence suggests that externalized anger is positively associated with symptoms of depression and anxiety (Bridewell & Chang, 1997). Additionally, hostility predicts increased physical and emotional burnout (Razani et al., 2014), and psychosomatic stress responses, which contribute to decreased quality of life (Smith, 1992). Indeed, researchers who examine heightened hostility in response to traumatic events report that the increased negative emotionality predicts increased distress and suboptimal long-term mental and physical health outcomes (Galatzer-Levy et al., 2013; Smith, 1992). Thus, although hostility may signal an individual to mobilize resources to confront a threat, increased levels of hostility and prolonged hostile reactions predict increased distress over time.

What remains unclear are how these trauma–hostility–distress processes work together, specifically alongside social support. There is abundant research on the direct role of social support in mitigating trauma responses and alleviating experienced negative emotion and global distress. However, there is a paucity of research that examines how social support modifies trauma’s interplay with hostility in predicting increased psychological distress. Altogether, the literature suggests that increased hostility in response to threat predicts worsened psychological outcomes such as increased stress, anxiety, and depression (Heponiemi et al., 2006). Luckily, literature also suggests that that greater perceived social support may weaken the pathway between initial traumatic reaction and hostility (Alradaydeh & Alorani, 2017; Jun et al., 2018). In this way, social support may serve as a robust buffer that modifies the emotional connections between initial trauma onset and subsequent worsened mental health outcomes.

## 4 | A PERSISTENT GLOBAL STRESSOR

Much work has investigated the impact of acute stressors on psychological distress, such as natural disasters and even the COVID-19 lockdown, but extending this literature by testing the mechanisms through which acute stressors predict distress has received less attention. We would predict the associations between social support and psychological distress to be especially prominent at the onset of a stressful event, when imposed lifestyle changes such as mandated masks, shuttered businesses, and distance-only learning, were particularly distressing. The traumatic impact at the onset of lockdown should operate similarly to how acute stress modifies the effects of support leading up to an acute, known stressor (Bolger et al., 2000; Shrout et al., 2010). Thus, although the COVID-19 pandemic is not singularly unique in terms of stress, it does provide an amplified context in which to study this generalized stress reactivity pathway.

As the damaging impacts of the pandemic and other global, potentially overwhelming threats (e.g., the monkeypox outbreak, climate change events, war conflict) continue to unfurl, researchers are more seriously investigating systems affected by seemingly distinct historical contexts as they relate to broader psychological theory. For example, Lahav (2020) explored exposure to previous trauma in a sample from Israel and found that among those previously exposed to traumatic events, stress from COVID-19 predicted increased PTSD, anxiety, and peritraumatic symptoms. There is a dearth of published work on the impact of persistent, large-scale stressors working in concert with common buffers to traumatic impact, such as perceived social support systems, and vitally, emotional reactions to threat. This gap in information is crucial to address because there is likely a host of individual differences in how people experience, emotionally react to, and address the challenges of overwhelming stressors. Considering postulations that there are inevitably other, arguably more life-changing global stressors to face us in the coming years, it is prudent to begin to understand how these processes work in tandem. Such research could provide information to help arm individuals to cope with current and future overwhelming stressors.

## 5 | PRESENT STUDY

**FIGURE 1:**
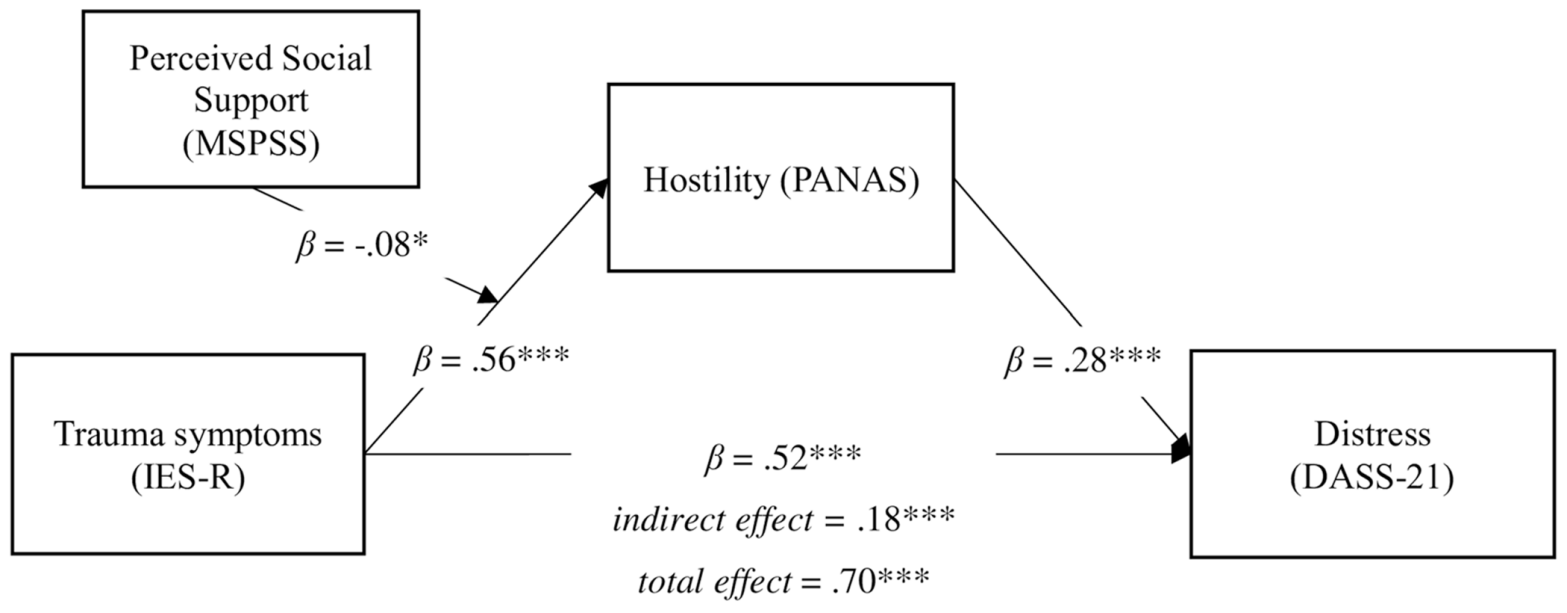
Results for moderated mediation model. DASS-21, Depression, Anxiety, and Stress Scales-21 item; IES-R, Impact of Event Scale-Revised; MSPSS, Multidimensional Perceived Social Support Scale; PANAS-Hostility, Positive and Negative Affect Schedule, Hostility subscale. ***p < 0.001, *p < 0.05.

We sought to extend the current literature by clarifying social support’s association with distress in reaction to COVID-19-related stress, namely via hostility. To do this, we surveyed a large, heterogeneous sample of individuals (*n* = 426) from a university setting (i.e., undergraduate and graduate students, faculty, administration, and support staff) shortly after the onset of the COVID-19 local lockdown in the final week of March 2020. By examining this cohort over the strictest period of local lockdown, we sought to understand how perceived social support would attenuate the effects of increased trauma-response symptoms on depression, anxiety, and stress, as mediated by hostility, during a time of disorientation and fear for many. Our analyses, in the form of a moderated mediation model (Figure 1), therefore focus on whether social support buffers against increased hostile reactions (Path a) and subsequent elevated depression, stress, and anxiety (i.e., psychological distress) in response to increased hostility (Path b) upon the widespread, systemic restrictions on personal and professional functioning.

### 5.1 | Hypotheses

**H1**. Severity of trauma symptoms, indexed by increased avoidance and hypervigilant attitudes and behaviors, will predict an increased hostility response (H1a), which will in turn lead to higher global psychological distress, indexed by a combined measure of depression, stress, and anxiety symptoms (H1b), yielding an overall indirect effect (H1c).

**H2**. Social support will attenuate the relationship between trauma symptoms and hostility (i.e., an interaction on Path a) such that those higher in perceived social support will experience a weaker indirect link between trauma and distress via hostility.

## 6 | METHODS

### 6.1 | Participants

Participants were recruited for the study using two approaches. First, the survey was posted to a large, Midwestern university website (hosted by Sona Systems) to complete for research credit. Students recruited via SONA were those enrolled in an undergraduate-level introductory Psychology course. This course is required across many colleges and majors, assisting in university-wide representation. Next, primarily nonundergraduate (i.e., faculty, staff, and graduate student) participants were recruited via an email that was sent on behalf of the research team to a variety of publicly available email lists associated with several colleges within the host university. These emails explained the broad purposes of the research and included a link to the study. Although undergraduates could gain access to the listserv email, this method was much less likely to recruit undergraduates. Individuals who had already completed the survey through Sona were actively discouraged from completing the survey through a listserv email. In total, 14 participants from the listserv group self-identified as undergraduates. Both surveys were programmed in Qualtrics. Data collection occurred from March 20 through March 27, 2020, via the research crediting system, and from March 24 to April 1, 2020 via the email link.

A total of 426 individuals participated in the survey, but we excluded individuals who demonstrated nonpurposeful responding or minimally complete responses (*n*_excluded_ = 18). Two attention check items were embedded within the survey that indicated a specific response to rate that item (e.g., “select ‘moderately’ for this item”). Participants were excluded from the analysis if they failed both of the attention checks (*n* = 8), took <5 min to complete the survey (*n* = 14), and/or clicked through <97% of the survey (*n* = 3; 97% completion indicated only that a participant clicked through to the last page of the survey because no answers were required). Thus, 408 participants were retained for analyses. Two hundred and eighteen participants were recruited via the email link and 208 participated for research credit. The majority of participants were female, White, heterosexual, and never married (full demographic characteristics of the sample are listed in Table 1). For further details on the study design and sample, see Balling et al. (2021). Answering demographic questions was not required.

### 6.2 | Measures

#### 6.2.1 | Trauma symptoms

The 22-item Impact of Event Scale-Revised (IES-R) is the most widely used self-report measure of traumatic stress (Creamer et al., 2003; Horowitz et al., 1979; Weiss & Marmar, 1997). It assesses posttraumatic experiences of intrusion, avoidance, and hyperarousal over the past week. The items feature a five-point Likert scale response ranging from 0 (“not at all”) to 4 (“extremely”). In our survey, we asked participants to consider questions, “with respect to the Coronavirus (COVID-19) outbreak.” Examples of items included, “I stayed away from reminders of it,” and, “I thought about it when I didnt’ mean to.” Items were summed to create subscales indexing hyperarousal, avoidance, and intrusion. Subscale scores were then summed to create a total scale indexing traumatic impact (e.g., Arnberg et al., 2012; Soroush et al., 2022). Research has indicated high levels of internal consistency for the full scale (*α* ≥ 0.95; Beck et al., 2008; Creamer et al., 2003). In the present sample, Cronbach’s *α* = 0.92 for the total IES-R score.

#### 6.2.2 | Hostility

The six-item hostility subscale of the widely-used and well-validated Positive and Negative Affect Schedule (PANAS) was administered to assess self-reported experiences of anger, hostility, irritability, scorn, disgust, and loathing during the past week (Crawford & Henry, 2004; Watson & Clark, 1994; Watson et al., 1988). Participants rated the emotions from 1 (“very slightly or not at all”) to 4 (“quite a bit”). Cronbach’s *α* is high in the extant literature, ranging from *α* = 0.86 to 0.90 for positive affect scales, *α* = 0.84 to 0.87 for negative affect scales, and the hostility subscale *α* > 0.75 (Harmon-Jones, 2004; Watson et al., 1988). Items from this scale were summed to create a scale for negative affect. In the present sample, Cronbach’s *α* = 0.83 for the total hostility score.

#### 6.2.3 | Social support

The 12-item Multidimensional Scale of Perceived Social Support (MSPSS) assesses perceived support from family, friends, and significant others (Bruwer et al., 2008; Zimet et al., 1988). This scale is designed to capture the multifaceted nature of perceived social support. Items include: “I can talk about my problems with my friends,” “My family really tries to help me,” and “There is a special person in my life who cares about my feelings.” Response options feature a Likert scale ranging from 1 (“very strongly disagree”) to 7 (“very strongly agree”). Item responses were summed and averaged across their respective subscales, which index social support received from friends, family, and significant others. The total score was obtained by averaging all items. Internal consistency has been reported previously at *α* = 0.88 for the full scale (Zimet et al., 1988) and a single, higher-order factor is well supported (Clara et al., 2003). In the present sample, we have strong internal consistency with Cronbach’s *α* = 0.92 for the total MSPSS score.

#### 6.2.4 | Distress

The Depression, Anxiety, and Stress Scales-21 Item (DASS-21) is a widely used tool that has evidenced validity in community settings and a variety of cultures (Antony et al., 1998; Lovibond & Lovibond, 1995; Tran et al., 2013). It is a self-report measure assessing symptoms of depression, anxiety, and stress over the past week. Response options range on a four-point Likert scale from 0 (“did not apply to me at all”) to 3 (“applied to me very much, or most of the time”). Examples of items include: “I felt that I had nothing to look forward to,” “I felt I was close to panic,” and “I found it difficult to relax.” Item responses were summed to create subscales that indexed stress, anxiety, and depression. Subscales were summed to generate a total score reflective of current distress, as is supported by extant literature (e.g., Henry & Crawford, 2005). In past research, the total score on the DASS-21 exhibits strong internal consistency (Cronbach’s *α* = 0.91–0.93; Henry & Crawford, 2005; Le et al., 2017). In the present sample, Cronbach’s *α* = 0.94 for the total DASS score.

## 7 | PROCEDURE

No directly identifiable information was collected from participants, so the study was approved for exemption from full review by the host university’s Institutional Review Board (IRB-2020-486). Upon accessing the survey, participants read the informed consent and indicated whether or not they consented to participate in the study. If they consented, they proceeded to the Qualtrics survey. The median time to complete the survey for included responses was 11 min, 45 s, with 95% of participants’ completion time under 54 min.

Participants who completed the survey through the Sona system were granted course credit. Those who accessed the survey through the email list had the opportunity to enter a drawing for one of three $40 Amazon gift cards available for each wave. Following survey completion, participants could follow a link to a separate Qualtrics survey and provide their email address to be entered into the raffle. The identifying information from the raffle entry was not linked in any way to the participants’ main survey data. Following data collection, the investigators used a random digit generator to select three winners and email each of them an electronic Amazon gift card.

## 8 | SENSITIVITY POWER ANALYSES

As our sampling strategy was to collect data from as many individuals as possible within the restricted time intervals we specified a priori, a traditional power analysis to determine the required number of individuals to achieve sufficient power for the set of effects of interest is not maximally useful. Instead, we used the strategy outlined by Lane and Hennes (2019) to conduct a series of power analyses that varied (a) the size of the sample we believed we could feasibly expect to collect, (b) the size of *a* and *b* path effects for trauma associated with hostility and hostility with distress, respectively, based on past literature, and (c) the size of the interaction effect by which support was hypothesized to attenuate the positive association between trauma and hostility, also informed by previous literature. In total, we examined 81 combinations across three levels each of sample size (*N* = 300, 1000, 1500), Path a effect size (*β* = 0.40, 0.50, 0.60), Path *b* effect size (*β* = 0.20, 0.30, 0.40), and interaction effect size (*β* = −0.05, −0.10, −0.15; see Table 2). The ranges for each were determined as follows:

- Given the public university-wide email listservs we were able to compile and the available introductory undergraduate participant pool completing studies for course credit, we conservatively anticipated that we could recruit approximately 300 responders. Thus, we selected a minimum sample of 300 and a maximum of 1500, at which any effect of substantive interest should be detectable.
- Previous research has demonstrated a strong link between current experiences of trauma and reports of anger, hostility, or aggression (*β* = 0.48, Orth & Wieland, 2006) and generally moderate associations between hostility-related reactions and psychological distress (*β* = 0.39, Balta et al., 2016). We used these reports as representative starting-point estimates and then reduced each to guard against possible effect size inflation in published studies.
- There is less work that examines the potential impact of social support on the link between experienced trauma and hostile aggression. Evidence suggests that social connection buffers traumatized individuals from experiencing angry/externalizing symptoms (*β* = −0.09, Jun et al., 2018). There is also more general evidence that the negative effects of both acute and chronic stress on emotional health/well-being are attenuated by objective and subjective perceptions of support availability as a direct effect (*β* = −0.42, Olstad et al., 2001). In light of these findings, we selected a small overall effect size and narrow range of possible interaction effect sizes based on the expected ordinal/synergistic interaction effect.

Based on our sample size and parameter specifications, the *a* path, *b* path, and overall *ab* indirect effect would achieve at least 90% power for all of our simulated parameter specifications. We do not discuss this further, as the observed effects were on the upper end of our hypothesized ranges, indicating near 100% power for each.

Power estimates for the interaction effect size of support on the trauma→hostility (*a* path) regression coefficient and the index of moderated mediation (*β*_Interaction_ × *β_b_*; *i*_M_) were nearly perfectly correlated (*r* > 0.96), so we focus on the more conservative *i*_M_. In either case, the effect size of the moderation effect was more impactful than either of the *a* or *b* paths’ direct effects. When the moderation effect was very small (*β* = −0.05) power was poor (<20%) for small sample sizes (*N* = 300), but leveled with conventional *p*-values (i.e., *p* = 0.05; Power = 50%) just above the midpoint in sample size range (*N* = 1000). At conventionally small effect sizes (*β* = −0.10), the power was generally poor for small sample sizes (*N* = 300), but excellent for larger sample sizes (*N* = 1000). Any interaction effect size greater than *β* = −0.15 would have adequate power for a sample size as small as *N* = 300.

Given the acquired sample size, the overall direct and indirect effect sizes can likely be incorporated as reliable estimates, in this sampling environment, for the associations between trauma, hostility, and distress. The associations with respect to how social support attenuates, or otherwise protects, the specific link between trauma and hostility, still requires more investigation. The current data and mapping of multivariate effect sizes in a moderated mediation model with respect to feasibility constraints lends credence to the sensitivity analysis process (Table 2, shaded area). That is, the power analyses could not predict what effect sizes (and *p* values) would eventually be observed; but they did effectively situate the observed effects in terms of what the collected data could have looked like and how drastically inferences could have changed with minor alterations in the observed model effects.

### 8.1 | Data analysis

Full data, including additional variables and sampling occasions, are available at osf.io/5prc3. All variables were approximately normally distributed (skew range = −1.01 to 0.99 across all variables). We first examined Pearson bivariate correlations among study variables (see Table 3). We then performed a single-level moderated mediation analysis to assess effects of trauma symptoms, hostility, and social support on distress. In this case, single-level mediation is appropriate because of the context that lockdown provided—we argue that COVID-related changes (including lockdown) constitute a natural temporal precedent. Therefore, when we asked participants to report on past week trauma (as related to the COVID-19 related changes) and subsequent hostility and global distress, we approximate a temporal model. This is also supported by research cited above that ongoing traumatic impact predicts increased emotional reactions (e.g., Lahav, 2020). Accordingly, we argue that given the context of data collection and theoretical models that support the existence of this process, single-level mediation is an appropriate application in this study.

First, we regressed depression, anxiety, and stress (DASS-21) onto trauma symptoms (IES-R; Path *c*) and hostility (PANAS hostility subscale; Path *b*), and regressed hostility onto trauma symptoms (IES-R; Path *a*). We also tested whether perceived social support (MSPSS) moderated the relationship between trauma symptoms and hostility (Path *a*). Lastly, indirect effects of trauma symptoms on distress via hostility were assessed and probed for effects of social support. We statistically adjusted for differences in reported hostility and distress by recruitment group to ensure any observed effects were not simply driven by a group difference (i.e., the Sona vs. listserv groups). Analyses were conducted in SPSS v. 28 (IBM Corp., 2021) using Hayes’ (2017) PROCESS macro.

## 9 | RESULTS

Psychological distress (i.e., depression, anxiety, and stress), trauma symptoms, and hostility were significantly positively correlated, whereas perceived social support was significantly negatively correlated with distress, trauma symptoms, and hostility (see Table 3).

### 9.1 | Impact of acute stress and the mitigating effect of support on current hostility and generalized distress in response to trauma

In line with Hypothesis 1, results revealed that traumatic impact from COVID-19 significantly predicted increased hostility (H1a; Path *a*; *β* = 0.562, SE = 0.040, *p* < 0.001) and hostility was associated with increased psychological distress (H1b; Path *b*; *β* = 0.315, SE = 0.039, *p* < 0.001), independent of trauma symptoms (Path *c*; *β* = 0.523, SE = 0.039, *p* < 0.001). There was also a significant main effect such that greater perceived social support was associated with reduced hostility (*β* = −0.172, SE = 0.041, *p* < 0.001). In line with these effects and H1c, there was evidence of a significant indirect effect of trauma on distress via hostility (H1c; *β*_Indirect_ = 0.177) at low, average, and high levels of perceived social support according to bias-corrected bootstrapped confidence intervals (CIs); 95% CIs [low; 0.07–0.25], [avg.; 0.11–0.22], and [high; 0.09–0.24]. There was also a significant main effect of sample group for distress only, such that participants from the Sona system group reported significantly lower distress scores than the listserv group (β = −0.087, SE = 0.031, *p* < 0.01).

In support of Hypothesis 2, perceived social support attenuated the main effect of traumatic impact on increased hostility (Path a interaction; *β* = −0.077, SE = 0.038, *p* = 0.042). Model results are presented graphically in Figure 1.^1^

## 10 | DISCUSSION

The present study examined the buffering effects of perceived social support on initial emotional reactions to an extreme, ongoing stressor. We aimed to test how efficacious perceived social support would be in ameliorating increased hostility and subsequent symptoms of depression and anxiety. Broadly, considering the nature of the threat that the pandemic and large-scale stressors like it pose, results suggest that leveraging one’s support system to manage emotional reactions and long-term mental health consequences may be a crucial way to cope.

Our primary results support broad theoretical frameworks regarding appraisal of threat and social support: increased hostility in response to threat yielded marked psychological distress, traumatic impact predicted increased psychological distress, and perceived social support served as a buffer between traumatic impact and increased hostile reaction. This buffer between traumatic impact and hostility attenuated the indirect influence of traumatic impact on subsequent increased psychological distress via hostility during the initial phase of lockdown. Critically, our findings are supported in the broader literature, where perceived social support has been found to attenuate the effects of stress on mental health outcomes of many sorts. For example, cross-sectional evidence suggests that stronger perceived social support is associated with reductions in the relationship between PTSD symptoms and elevated stress in women exposed to sexual violence (Catabay et al., 2019) and longitudinal investigations indicate that perceived social support may: ameliorate the intensity of depression symptoms across adolescence (Heponiemi et al., 2006), attenuate general mental health distress in adults (Olstad et al., 2001), and reduce distress in soon-to-be parents throughout the transition to parenthood (Hughes et al., 2020). Others have demonstrated that perceived social support and other related protective factors, such as living with a romantic partner, attenuate anxiety, and threats to long-term mental health related to COVID-19 (Fernández et al., 2020).

Overall, we observed the proposed indirect mechanism of perceived social support buffering against increased feelings of hostility that, in turn, increased symptoms of depression, anxiety, and stress for individuals. Our results are consistent with the idea that perceived social support bolsters one’s sense of self-efficacy and imparts a sense that others close to the individual are available to “share the burden” (Norris & Kaniasty, 1996). In turn, this shield helps curb downstream effects of increased hostility that otherwise appear to extrapolate, including increased daily stress and symptoms of anxiety and depression that contribute to long-term patterns and potentially clinical levels of psychopathology (Catabay et al., 2019; Heponiemi et al., 2006). In an age of increased online presence that can replace in-person interactions, it is important to note that preliminary evidence suggests that online social support still reduces symptoms of depression and distress (Cole et al., 2017). As we continue to face global stressors (e.g., global warming, Hennes et al., 2016; social inequity, Reese et al., 2014), methods to curb the detrimental effects of trauma and hostility on distress (i.e., social support) become increasingly important and necessary.

## 11 | LIMITATIONS

Curiously, our data displayed group-specific effects, such that individuals recruited via Sona reported lower distress than the mixed listserv group. This detail may be explained by differences in responsibility and environmental demands. Specifically, all participants were sampled during the week of spring recess for the university, when all faculty, staff, administrative professionals, and most graduate students were transitioning course material from mostly in-person to a modality delivered fully online. Concurrently, many undergraduate students, while exposed to the same threat of COVID-19 lockdown, had just finished midterm exams and were on an academic break. Although we are not implying that the majority of the undergraduate group did not shoulder substantial burden, it is worth consideration that the workloads between the two groups varied and potentially contributed to the differences in overall reported distress. For example, many of the respondents in the listserv group were university employees (61%), were married and/or living with a partner (62%), and/or reported that they faced additional immediate stressors such as increased childcare responsibilities due to the pandemic (30%). In short, beyond academic considerations, there is evidence of differing levels of family impact across the two groups in our sample that could have contributed to this difference.

A further limitation of our study is the lack of diversity within the sample. Previous research has found that individuals from racial and sexual minoritized groups face increased and different stressors from groups that have majority status (Meyer, 2003). Moreover, previous literature suggests that perceptions of racism can influence the effect of perceived social support on stress (Clark, 2003). For example, in Black participants who reported high levels of perceived racism, social support was related to increased stress, whereas those who reported lower amounts of perceived racism evidenced lower stress in the context of greater social support (Clark, 2003). These results may differ from our findings and demonstrate the need for a more diverse sample in future studies.

Importantly, we did not examine an array of emotional reactions to the trauma symptoms, which could have clarified the unique role of hostility—relative to other negatively-balanced emotions, such as fear or sadness—in response to an overwhelming threat. However, we maintain that the mechanistic role of hostility to threat remains a vital piece to understanding reactions to threat (MacLaren et al., 2010).

Additionally, three important design limitations should be noted. To preserve anonymity and bolster confidence that their responses would not be tracked during data collection, we elected to exclude requesting age of the participants, although this information could have assisted in proxying age cohort effects and probing for differences among those cohorts. Next, we want to acknowledge that the cross-sectional nature of the study precludes our ability to draw causal inferences, particularly, as there are likely bidirectional associations among traumatic stress, global distress, and hostility levels. While it is possible that increased traumatic stress preceded this study, our questions assessing traumatic impact asked participants to report on “how distressing each difficulty has been … during the past week with respect to the Coronavirus (COVID-19) outbreak.” Although imperfect, we hope that this can lend some credence to our argument that traumatic impact was driving symptoms, though we acknowledge that this does not create temporal precedence and thus limits our ability to comment on causality.

Finally, data collection was staggered (i.e., between Sona and listserv participants). Therefore, responses may not be temporally identical and, indeed, are partially confounded with recruitment mode. Ideally, data collection could have taken place at the same time for each recruitment type, but the prioritization of generating a sufficient sample size limited this effort. Further, timing was staggered to optimize response rates of each recruitment group, so as to avoid particularly hectic timing for either group (e.g., we tried to capture responses from undergraduates after midterms and we collected from the listserv after break considering the transition to online learning). Fortunately, our intent was to study this phenomenon in the context of a large-scale stressor, rather than study the specific effects of COVID-19 on participants’ hostility and stress. Thus, while the timeframe introduced non-negligible variability, our findings do not rely specifically on this pandemic.

## 12 | STRENGTHS

This study also featured several strengths. These include a more inclusive sample than a typical study from a university setting; instead of only recruiting students seeking academic research credit for introductory psychology courses, we also recruited undergraduates, graduate students, faculty, and staff from a variety of colleges across campus. Next, we specified our hypotheses and proposed pathway *a priori* and pre-registered this study and its hypotheses. In combination with our sensitivity analysis and the theoretical basis that informed our hypotheses, we provide compelling evidence for the existence of our effects. Further, data acquisition occurred early in the pandemic’s progression in the United States. Specifically, data collection launched as lockdown began and served as an ideal testing bed for the purportedly robust buffering effect of perceived social support in the face of widespread crisis. Indeed, by testing a specific theoretical model and including potential moderators to the relationships between distress and mental health outcomes, we were able to expand literature that examines the impact of COVID-19, lockdown, and--most crucially--significant stressors in general (e.g., Tang et al., 2020; Wang et al., 2020). Additionally, as this study offers a look into the beginning stages of a global pandemic, it is our hope that it will be particularly beneficial in understanding how early responses to considerable threat inform theory on psychological reactivity and sustained psychological difficulties for subsequent health crises (e.g., the current U.S. Monkeypox outbreak [2022-2023]).

## 13 | CONCLUSION

Results from the present study are of particular relevance to the literature on emotion, coping, and perceived social support. Specifically, we identified one historically well-studied path by which individuals can shield themselves from the pernicious effects of trauma that threaten long-term mental health consequences. Our findings suggest that to the extent individuals are succumbing to threat, as evidenced by increased trauma symptoms, they are likely to experience increased hostility and symptoms of depression, stress, and anxiety. Importantly, though, higher perceived social support attenuates the effects of traumatic impact on hostility. That is, the degree to which an individual orients towards hostility when threatened is in part determined by whether they perceive having a support network to help shield them from harm. We have preliminary evidence that people who report particularly high levels of trauma in response to acute, global stressors also report the highest levels of hostility, but that perceived social support may serve as a robust protective factor for these individuals.

Although our sample was conducted in a university setting and is thus limited in terms of generalizability, the participants themselves represent a relatively heterogeneous, individualistic population. Given these characteristics, we assert that the findings merit consideration for how they may extrapolate to more general populations. Further, our results align with and extend existing social support literature with respect to unexpected, acute, and large-scale stressors. By identifying and connecting with one’s support system of close individuals and strengthening natural social supports, individuals may help one another to withstand the storm.

## Figures and Tables

**TABLE 1:** Demographic characteristics of total sample included in analyses (*n* = 408).^a^

Variable	Total sample		Sona (*n* = 208)		Listserv (*n* = 218)	
	*n*	%	*n*	%	*n*	%
Sex assigned at birth						
Female	295	72.3	115	59.9	180	83.3
Male	109	26.7	74	38.5	35	16.2
Intersex	1	0.2	1	1.0	1	0.2

Gender identity						
Female	298	73.0	118	61.5	180	83.3
Male	107	26.2	72	37.5	35	16.2
Other	1	0.8	0	–	1	0.5

Race						
White	297	72.8	122	63.5	175	81.0
Asian	64	15.7	43	22.4	21	9.7
Latinx	18	4.4	11	5.7	7	3.2
Black	12	2.9	8	4.2	4	1.9
Other	11	2.7	5	2.6	6	2.8

Sexual orientation						
Straight/heterosexual	364	89.2	170	88.5	194	89.8
Bisexual	21	5.1	12	6.3	9	4.2
Gay or lesbian	11	2.7	4	2.1	7	3.2
Other	3	0.7	1	0.5	2	0.9

Relationship status						
Never married	238	58.3	173	90.1	65	30.1
Married	106	26.0	0	–	106	49.1
Living with partner	28	6.9	4	2.1	24	11.1
Divorced	11	2.7	0	–	11	5.1
Widowed	3	0.7	0	–	3	1.4
Separated	2	0.5	0	–	2	0.9

Education						
Some college	161	27.2	146	76.0	15	6.9
Completed graduate school	111	27.2	0	–	111	51.4
Some graduate school	61	15.0	0	–	61	28.2
Graduated HS/received GED	39	9.6	36	18.8	3	1.4
Graduated 4-year college	33	8.1	8	4.2	25	11.6
Graduated 2-year college	1	0.2	1	0.5	0	–

University affiliation^b^						
Undergraduate student	203	49.8	189	98.4	14	6.5
Faculty	60	14.7	3	1.6	57	26.4
Staff	66	16.2	3	1.6	63	29.2
Graduate student	89	21.8	2	1.0	87	40.3
University alumni	17	4.2	0	–	17	7.9
Administration	6	1.5	1	0.5	5	2.3
University parent	3	0.7	0	–	3	1.4

a There are missing data in each category (sex, *n* = 3; gender, *n* = 3; race, *n* = 6; orientation, *n* = 9; relationship, *n* = 20; education, *n* = 2).

b Participants were instructed to select all that applied; therefore, some values sum to >100%.

**TABLE 2:** Power estimates for the IES-by-support interaction and index of moderated mediation as a function of sample size and *a*, *b*, and interaction effect sizes.

Effect size			Power					
			*N* = 300		*N* = 1000		*N* = 1500	
*β*_Interaction_	*β_a_*	*β_b_*	*β*_Interaction_	*i*_M_	*β*_Interaction_	*i*_M_	*β*_Interaction_	*i*_M_
−0.05	0.40	0.20	17%	8%	44%	41%	58%	55%
		0.30	17%	14%	44%	43%	58%	57%
		0.40	17%	15%	44%	44%	58%	57%
	0.50	0.20	18%	8%	49%	44%	63%	61%
		0.30	19%	14%	49%	47%	63%	62%
		0.40	18%	17%	49%	48%	63%	63%
	0.60	0.20	20%	8%	54%	49%	71%	68%
		0.30	20%	16%	55%	53%	71%	70%
		0.40	20%	18%	55%	54%	71%	70%

−0.10	0.40	0.20	49%	32%	94%	93%	99%	99%
		0.30	49%	45%	94%	93%	99%	99%
		0.40	49%	47%	94%	93%	99%	99%
	0.50	0.20	54%	33%	96%	95%	99%	99%
		0.30	54%	49%	96%	96%	99%	99%
		0.40	54%	52%	96%	96%	99%	99%
	0.60	0.20	61%	35%	97%	97%	100%	100%
		0.30	61%	54%	97%	97%	100%	100%
		0.40	61%	58%	97%	97%	100%	100%

−0.15	0.40	0.20	84%	66%	100%	100%	100%	100%
		0.30	84%	81%	100%	100%	100%	100%
		0.40	84%	83%	100%	100%	100%	100%
	0.50	0.20	88%	68%	100%	100%	100%	100%
		0.30	88%	85%	100%	100%	100%	100%
		0.40	88%	86%	100%	100%	100%	100%
	0.60	0.20	93%	68%	100%	100%	100%	100%
		0.30	93%	90%	100%	100%	100%	100%
		0.40	94%	92%	100%	100%	100%	100%

*Note*: Shaded region indicates approximate power for the observed effects based on estimates closest to final sample (*N* = 408).

Abbreviations: *β_a_*, standardized effect for the IES→Hostility path in the moderated mediation model; *β_b_*, standardized effect for the Hostility→DASS path in the moderated mediation model; *β*_Interaction_, standardized effect for moderation of βa; the interaction between IES and support; DASS, Depression, Anxiety, and Stress Scale; IES, Impact of Event Scale; *i*_M_ is the index of moderated mediation (*β*_Interaction_ × *β*_*b*_).

**TABLE 3:** Descriptive statistics and correlations among global distress, social support, trauma symptoms, and hostility measures.

		*M* (SD)	1.	2.	3.	4.
1.	DASS-21	29.29 (24.95)	–	−0.25***	0.72***	0.63***
2.	MSPSS	5.63 (1.05)		–	−0.10*	−0.23***
3.	IES-R	25.10 (15.64)			–	0.59***
4.	PANAS-H	12.05 (4.60)				–

Abbreviations: DASS-21, Depression, Anxiety, and Stress Scales- 21 item total scale score; IES-R, Impact of Event Scale-Revised total scale score; MSPSS, Multidimensional Perceived Social Support Scale total scale score; PANAS-H, Positive and Negative Affect Schedule–Hostility subscale score.

*** *p* < 0.001;

* *p* < 0.05.

## Data Availability

The data that support the findings of this study are openly available in Open Science Foundation at https://osf.io/5prc3.

## Footnotes

1 To detect differences in patterns among the global distress subscales (i.e., depression, anxiety, and stress subscales), we performed three identical analyses to the primary analysis, with each DASS-21 subscale as outcome. Each subscale performed nearly identically to the model with the global distress score, with only slightly attenuated estimates on Paths *b* and *c*. In sum, we have strong evidence to suggest that each subscale functions similarly to the total score in our model.
